# Backache

**Published:** 1900-04-28

**Authors:** 


### BACKACHE.

Backache is one of the commonest of complaints; and although, of course, only a symptom is one of such persistence, and one which intrudes itself so forcibly upon the attention of the sufferer, that when it is at all pronounced it is apt to be regarded, by the patient at least, as the main point, the thing above all else to the relief of which treatment should be directed. It behoves us, then, to be well aware how varied are the causes by which it may be produced. In a paper recently read before the New Cross Medical Society, Mr. Harding Freeland pointed out how often this symptom is an indication of the existence of some disorder in the rectum. As he says, any morbid sensation referred to that portion of the back which corresponds to the posterior surface of the sacrum is frequently due to reflex irritation having its origin in the rectum, and even when it is associated with symptoms referable to the other pelvic organs—such as the bladder and the uterus—the rectum will not infrequently be found to be the organ primarily at fault. This dull, weary, aching pain over the sacrum is of a peculiarly annoying character and often prevents a man concentrating his mind upon his work. In such cases the presence of scybalous stools with viscid mucus may give the key to the situation, pointing, as it does, to chronic rectal catarrh—often only a part of a general catarrhal condition of the whole gastro-intestinal tract—which has become complicated by the lodgment of scybala. The treatment is to remove the scybala, to secure an efficient and regular action of the bowels, to relieve the catarrh, and to improve the general health. But this form of backache is not always due to such simple causes, for it may in many cases be traced to more or less serious organic disease—for example, to piles, ulcer, or cancer. In the latter cases it is especially important not to overlook the warning backache which sometimes so long precedes the more definite symptoms, or to treat it as weakness, rheumatism, and so forth. Backache is also in some cases associated with other signs of bladder disturbance, such as frequency of micturition or retention of urine, and in women by vaginal discharge, and yet the whole thing may be due to an impaction of fæces in the rectum. The association of backache and leucorrhœa may also result from piles. The moral is that the investigation of no case of backache can be considered complete until the condition of the rectum has been ascertained.

